# PE-MT: A Perturbation-Enhanced Mean Teacher for Semi-Supervised Image Segmentation

**DOI:** 10.3390/bioengineering12050453

**Published:** 2025-04-25

**Authors:** Wenquan Wang, Zhongwen Li, Xiaoyun Zhang, Gaoqiang Jiang, Yabo Wu, Shuchen Yu, Bihan Tian, Mingzhe Hu, Xiaomin Xu, Wencan Wu, Quanyong Yi, Lei Wang

**Affiliations:** 1 Wenzhou Third Clinical Institute Affiliated to Wenzhou Medical University, The Third Affiliated Hospital of Shanghai University, Wenzhou People’s Hospital, Wenzhou 325041, China; wzwwq6768@163.com (W.W.); hmz13858890069@163.com (M.H.); hongweilong@wmu.edu.cn (X.X.); 2 Ningbo Key Laboratory of Medical Research on Blinding Eye Diseases, Ningbo Eye Institute, Ningbo Eye Hospital, Wenzhou Medical University, Ningbo 315040, China; li.zhw@wmu.edu.cn; 3 The Business School, The University of Sydney, Sydney 2006, Australia; sherryz1888@163.com; 4 National Engineering Research Center of Ophthalmology and Optometry, Eye Hospital, Wenzhou Medical University, Wenzhou 325027, China; jgaoqiang@outlook.com (G.J.); yupaopao021@gmail.com (S.Y.); tianbh711@163.com (B.T.); wuwencan118@163.com (W.W.); 5 School of Biomedical Engineering and Imaging Sciences, King’s College London, London WC2R 2LS, UK; yabocool@gmail.com; 6 National Clinical Research Center for Ocular Diseases, Eye Hospital, Wenzhou Medical University, Wenzhou 325027, China

**Keywords:** medical images, semi-supervised segmentation, exponential moving average, uncertainty map, mean teacher

## Abstract

The accurate segmentation of medical images is of great importance in many clinical applications and is generally achieved by training deep learning networks on a large number of labeled images. However, it is very hard to obtain enough labeled images. In this paper, we develop a novel semi-supervised segmentation method (called PE-MT) based on the uncertainty-aware mean teacher (UA-MT) framework by introducing a perturbation-enhanced exponential moving average (pEMA) and a residual-guided uncertainty map (RUM) to enhance the performance the student and teacher models. The former is used to alleviate the coupling effect between student and teacher models in the UA-MT by adding different weight perturbations to them, and the latter can accurately locate image regions with high uncertainty via a unique quantitative formula and then highlight these regions effectively in image segmentation. We evaluated the developed method by extracting four different cardiac regions from the public LASC and ACDC datasets. The experimental results showed that our developed method achieved an average Dice similarity coefficient (DSC) of 0.6252 and 0.7836 for four object regions when trained on 5% and 10% labeled images, respectively. It outperformed the UA-MT and can compete with several existing semi-supervised learning methods (e.g., SASSNet and DTC).

## 1. Introduction

The accurate segmentation of medical images, such as computed tomography and magnetic resonance imaging, is pivotal for clinical applications ranging from disease diagnosis to treatment planning [1,2,3]. Such segmentation can assist with the detection of regions of interest (ROIs) in medical images and the assessment of the morphological characteristics of the regions. A large number of image segmentation methods have been proposed in the last decade [4,5], and most of them are based on deep learning technology [6]. These deep learning-based methods have achieved remarkable segmentation performance in fully supervised settings, but their performance heavily relies on large-scale labeled image data. Manual pixel-level labeling, however, is labor-intensive and time-consuming, especially for medical images where expert knowledge is required. This greatly restricts the applications of these deep learning-based segmentation methods [7,8,9]. To alleviate the scarcity of labeled data, different deep learning technologies have been proposed to take advantage of unlabeled image data, such as self-supervised learning [10,11], semi-supervised learning [12,13,14], and weakly supervised learning [15,16]. Among these technologies, semi-supervised learning (SSL) has emerged as a promising paradigm, leveraging both labeled and unlabeled data to enhance model generalization.

Many SSL methods have been proposed to fully integrate a small amount of labeled data and a large amount unlabeled data for accurate image segmentation. For example, Tarvainen et al. [14] proposed the classical mean teacher (MT) method by using the exponential moving average (EMA) scheme to align a student model and a teacher model, which were enforced to have consistent predictions for unlabeled images. Wang et al. [17] improved the MT by introducing a unique model-level residual perturbation and an exponential Dice (eDice) loss. Yu et al. [18] proposed an uncertainty-aware mean teacher (UA-MT) method by using entropy uncertainty maps to filter out unreliable boundary prediction by the teacher model. Sukesh et al. [19] improved the UA-MT by using a pre-trained denoising auto-encoder (DAE) to generate uncertainty maps and reduce the overhead of computational resources. Li et al. [20] developed a multi-task deep learning network and introduced an adversarial loss between the predicted signed distance maps (SDM) of labeled and unlabeled data. Luo et al. [21] proposed a dual-task consistency semi-supervised method by explicitly establishing task-level regularity. Shi et al. [22] utilized different decoders to generate certainty and uncertainty object regions and help a student network to learn from them with different network weights. These semi-supervised segmentation methods have the potential to handle various medical images and obtain promising applications, but they may suffer from relatively large segmentation errors (especially in object boundary regions). This is probably due to the fact that (1) the EMA can lead to a tight coupling between the network weights of the student and teacher models, making the two models have very similar predictions for unlabeled images and thus suppressing the learning potential of the student model from the predictions of the teacher model. (2) The boundary regions of target objects are not effectively processed by the student and teacher models or existing uncertainty strategies in these semi-supervised methods, thus leading to relatively large segmentation errors.

In this paper, we developed a novel semi-supervised learning method (called PE-MT) for accurate image segmentation based on the UA-MT by introducing a perturbation-enhanced EMA (pEMA) and a residual-guided uncertainty map (RUM) to overcome the drawbacks of the traditional EMA and entropy uncertainty map (EUM). The pEMA was used to provide proper network weights for both the student and teacher models and alleviate the coupling effect between them via the modulus operator, while the RUM was used to highlight the unreliable prediction in the boundary regions of target objects leveraging a unique uncertainty quantitative formula and force the student model to focus on the other regions. With the two components, our developed method is expected to have reasonable potential to handle medical images with varying modalities and obtain promising segmentation performance, as compared to the UA-MT and several semi-supervised methods.

## 2. Method

### 2.1. Scheme Overview

**Figure 1:**
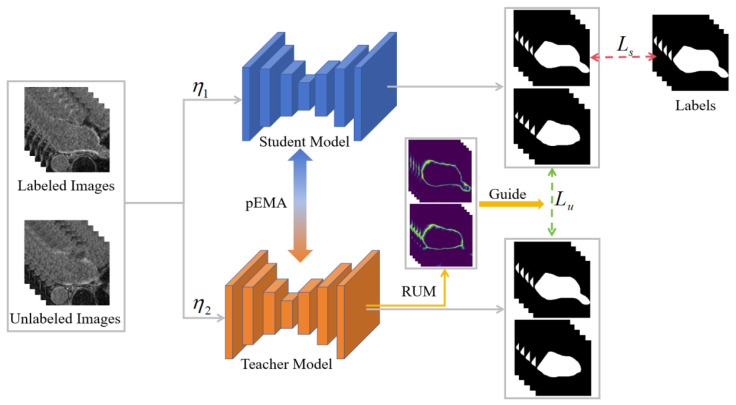
Overview of the developed semi-supervised segmentation method based on the UA-MT by introducing the unique PEMA and RUM schemes.

Figure 1 shows the developed semi-supervised segmentation method by introducing the pEMA and RUM to improve the learning potential of the teacher and student models in the available UA-MT. The two models share the same network backbone (e.g., U-Net or V-Net), but their network weights are updated through distinct mechanisms. Specifically, the teacher’s weights are obtained using the student’s weights from different training steps through the pEMA, which not only enables the teacher model to capture the information learned by the students but also reduces the coupling between the teacher and student models. With the obtained weights, the teacher model can generate a prediction for each unlabeled image. These predictions are then thresholded by the RUM to filter out unreliable regions and used as pseudo-labels for unlabeled images. With these pseudo-labels, the student model can extract a great number of discriminative features from a small number of labeled images and a large number of unlabeled images for segmentation purposes, leveraging the supervised and unsupervised losses. Minimizing these two losses enables the student and teacher models to achieve very similar segmentation performance.

### 2.2. Semi-Supervised Segmentation

To minimize the supervised and unsupervised losses, we trained the developed semi-supervised method based on a training set consisting of N labeled images and M unlabeled images. The labeled and unlabeled images can be represented by $S_{l}={\left\{(x_{i},y_{i})\right\}}_{i=1}^{N}$ and $S_{u}={\left\{x_{i}\right\}}_{i=1}^{M}$, respectively, where $x_{i}$ and $y_{i}\in R^{H\times W\times D}$ denote the involved image and label (i.e., ground truth) with specific dimensions of height H, width W, and depth D. With the images and labels, the total loss function for our developed method can be defined as follows:

(1) $$L_{total}=\sum_{i=1}^{N}L_{s}(p_{i}^{s}(x_{i},\theta ),y_{i})+\lambda \sum_{i=1}^{N+M}L_{u}(p_{i}^{s}(x_{i},\theta ,\eta_{1}),p_{i}^{t}(x_{i},\theta^{*},\eta_{2}))$$

(2) $$\begin{array}{cc} L_{s} & =CE(p_{i}^{s}(x_{i},\theta ),y_{i})+Dice(p_{i}^{s}(x_{i},\theta ),y_{i})\\ & =-\frac{1}{\left|\Omega \right|}\sum_{\Omega }y_{i}\log\left(p_{i}^{s}(x_{i},\theta )\right)+1-\frac{\sum_{\Omega }2p_{i}^{s}(x_{i},\theta )y_{i}}{\sum_{\Omega }p_{i}^{s}(x_{i},\theta )+\sum_{\Omega }y_{i}} \end{array}$$

(3) $$L_{u}=\frac{1}{\left|\Omega \right|}\sum_{\Omega }|p_{i}^{s}(x_{i},\theta ,\eta_{1})-p_{i}^{t}(x_{i},\theta^{*},\eta_{2}){|}^{2}$$

where θ and $\theta^{*}$ denote the network weights of the student and teacher models, $\eta_{1}$ and $\eta_{2}$ denote small random noises added to labeled and unlabeled images, respectively. $p_{i}^{s}(x_{i},\theta )$, $p_{i}^{s}(x_{i},\theta ,\eta_{1})$, and $p_{i}^{t}(x_{i},\theta^{*},\eta_{2})$ indicate the predictions of image $x_{i}$ obtained by the student and teacher models under small random noises, $\eta_{1}$, and $\eta_{2}$, respectively. $L_{s}$ is the supervised loss, comprising the cross entropy (CE) and Dice loss functions [23], and |Ω| denotes the number of pixels in the image domain Ω. $L_{u}$ is the unsupervised losses and used to assess the consistency between predictions $p_{i}^{s}(x_{i},\theta ,\eta_{1})$ and $p_{i}^{t}(x_{i},\theta^{*},\eta_{2})$ based on the pixel-wise mean-squared error (MSE). λ is a scalar factor used to keep the balance between $L_{s}$ and $L_{u}$ and is often set to $\lambda \left(t\right)=0.1e^{-5{\left(1-t/t_{\max}\right)}^{2}}$ according to previous studies [14,17], where t and $t_{\max}$ denote the current and maximum iteration number during network training, respectively. For simplification, the predictions of image $x_{i}$ obtained by the student and teacher models are represented by $p_{i}^{s}$ and $p_{i}^{t}$, respectively.

### 2.3. The pEMA

The pEMA was derived from the EMA and used to provide a small weight perturbation for the student model so that it could obtain better accuracy and generalization capability in image segmentation. The EMA and pEMA can be separately given by

(4) $$\theta_{t}^{*}=\alpha \theta_{t-1}^{*}+(1-\alpha )\theta_{t}$$

(5) $$\left\{\begin{array}{c} \theta_{t}^{*}=\alpha \theta_{t-1}^{*}+(1-\alpha )\theta_{t}\\ \theta_{t}=\theta_{t}+\beta \mathrm{mod}(\theta_{t}^{*},\theta_{t}) \end{array}\right.$$

where $\theta_{t}^{*}$ denotes the network weight of the teacher model obtained by the EMA based on the student’s weight $\theta_{t}$ at the training step t. α and β are two different scalar factors. $\mathrm{mod}\left(\cdot \right)$ is the element-wise modulus operator. Based on the two formulas, it can be seen that in the original EMA, the student’s weight was obtained on a small number of labeled images and the teacher’s weight was merely derived from the student’s weights at different training steps. This calculation scheme made the teacher’s weight very similar to the student’s weight, thus limiting the efficient utilization of unlabeled images. Conversely, in the pEMA, the student model is updated based not only on labeled data at the current training step but also on a given residual perturbation between the student and teacher weights via the modulus operator. This can, to some extent, make the two models have different network weights and thus alleviate the coupling effect between them. On the other hand, the residual perturbation was closely associated with both the student and teacher weights and adaptively changed as the network was trained, which gave the pEMA the potential to improve the segmentation performance of the two models.

### 2.4. The RUM

The RUM was constructed based on multiple forward passes [24] of the teacher model under random image-level perturbation (e.g., dropout and noise) to show its prediction reliability for desirable objects depicted on unlabeled images. It can be given by

(6) $$RUM\left(\upsilon \right)=\sum_{c=1}^{C}\left({\bar{p}}_{c}^{{\left(1-{\bar{p}}_{c}\right)}^{\upsilon }}-{\bar{p}}_{c}\right)$$

(7) $${\bar{p}}_{c}=\frac{1}{K}\sum_{k=1}^{K}p_{k,c}^{t}$$

where $p_{k,c}^{t}$ denotes the k-th forward pass of the teacher model for class c in unlabeled image x, and K and C are the total number of forward passes and classes. υ is a scalar coefficient and used to adjust the mean prediction probability ${\bar{p}}_{c}$ in the RUM. With the unique quantitative formula, our uncertainty map had better capability to locate image regions with high uncertainty (especially boundary regions of target objects) and highlight the prediction unreliability for these regions, as compared to the original entropy uncertainty maps (EUMs) in the UA-MT, which was widely used in previous studies and defined as follows:

(8) $$EUM=-\sum_{c=1}^{C}{\bar{p}}_{c}\log\left({\bar{p}}_{c}\right)$$

**Figure 2:**
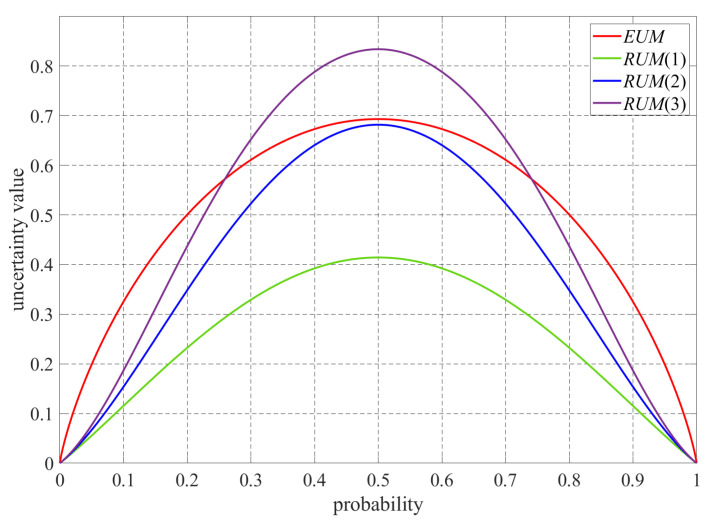
Differences between the RUM and EUM based on the prediction probability of a voxel/pixel for all the classes, where the parameter υ was set to 1, 2, and 3, respectively.

Figure 2 illustrates the differences between the RUM and EUM based on the prediction probability p of a pixel for a segmentation task with a class number of 2 (i.e., p for the desirable region, and (1−p) for the background). According to Equations (7) and (8), the RUM and EUM have similar quantization curves and reach their corresponding maximum uncertainty value at a probability of 0.5 since a probability of 0.5 is often used to decide whether a pixel belongs to object regions or not in deep learning. However, the RUM has a larger maximum at a probability of 0.5 and its curve has a steeper slope, suggesting that our RUM can quickly and accurately locate uncertainty prediction regions and then exclude these regions in the unsupervised loss.

Based on the introduced RUM, we can enhance the consistency between the predictions of the student and teacher models for unlabeled images by filtering out image regions with high uncertainty in the unsupervised loss:

(9) $$L_{u}=\frac{\sum_{\Omega }I\left(RUM\left(\upsilon \right)<\tau \right)|p^{s}-p^{t}{|}^{2}}{\sum_{\Omega }I\left(RUM\left(\upsilon \right)<\tau \right)}$$

where τ is a given uncertainty threshold and assigned to $\left(0.75+0.25e^{-5(1-t/t_{\max})})\log(2\right)$ in the UA-MT, and I is a member function.

## 3. Experiments and Results

### 3.1. Dataset and Evaluation Metrics

**Figure 3:**
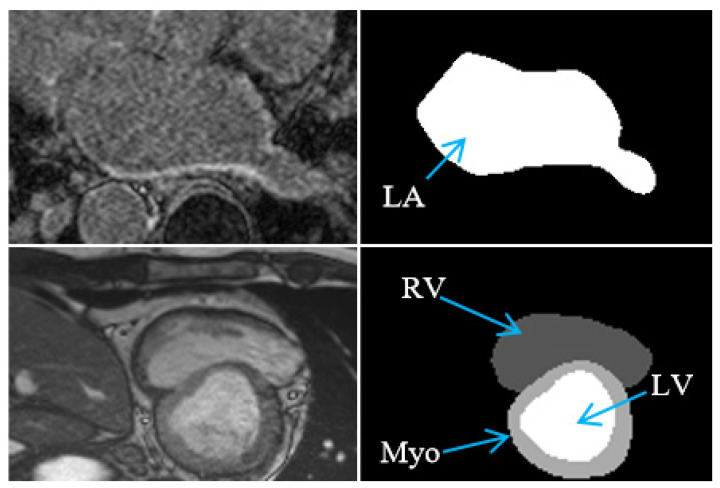
Illustration of images and labels in the LASC (**top row**) and ACDC (**bottom row**) datasets, respectively, where LA, Myo, LV, and RV denote the left atrium, myocardium, and left and right ventricles, respectively.

In this study, we used the Left Atrial (LA) Segmentation Challenge (LASC) dataset [25] and the Automated Cardiac Diagnosis Challenge (ACDC) dataset [26] to validate the developed method. The LASC dataset consists of 100 3D gadolinium-enhanced MRI scans (GE-MRIs) and their corresponding segmentation labels, both of which have an isotropic resolution of 0.625 × 0.625 × 0.625 mm^3^. These GE-MRIs were normalized to zero mean and unit variance and divided into 80 scans for network training and 20 scans for performance validation, following previous studies [18]. The ACDC dataset contains both end-diastolic and systolic-phase short-axis cardiac cine-MRI scans of 100 patients and their corresponding segmentation masks for three different tissue regions, including left ventricle (LV), myocardium (Myo), and right ventricle (RV). These data were divided into 70 and 30 patients’ scans for network training and validation, respectively. Because of the large spacing between short-axis slices and the possible inter-slice shift caused by respiratory motion, we used U-Net to segment each slice separately, as recommended by previous studies [27]. Figure 3 illustrates the images and their corresponding labels from the LASC and ACDC datasets.

We used the available V-Net [8,18] and U-Net [7] as backbone networks for LA and cardiac segmentation, respectively, and assessed their performance [28] leveraging the Dice similarity coefficient (DSC), Jaccard coefficient (JAC), 95% Hausdorff Distance (HD), and average surface distance (ASD), all of which were available in the MedPy library (https://github.com/loli/medpy) (assessed on 27 November 2024) and defined as follows:

(10) $$DSC=\frac{\sum_{\Omega }2py}{\sum_{\Omega }\left(p+y\right)}$$

(11) $$JAC=\frac{\sum_{\Omega }py}{\sum_{\Omega }\left(p+y-py\right)}$$

(12) $$HD=\max\left(hd\left(p,y\right),hd\left(y,p\right)\right)$$

(13) $$ASD=\frac{1}{\left|S(p)\right|}\left(\sum_{a\in S\left(p\right)}\underset{b\in S\left(y\right)}{\min}||a-b||\right)$$

where p and y denote the prediction of a given image and its corresponding label, respectively. $S\left(\cdot \right)$ is the set of surface voxels/pixels in an image. $\left\|a-b\right\|$ is the distance from point a to point b. $hd\left(p,y\right)=\underset{b\in S\left(y\right)}{\max}\left(\underset{a\in S\left(p\right)}{\min}||a-b||\right)$ is the directed HD from p to y. The DSC and JAC metrics are scored from 0 to 1, where higher values denote better segmentation accuracy. Conversely, ASD and HAD are distance-based metrics (measured in pixels), where values start above 0, and lower values correspond to smaller segmentation errors.

### 3.2. Implementation Details

We implemented the developed method via PyTorch (version 1.9.1) on a platform with an NVIDIA GeForce RTX 2080 SUPER GPU for two different segmentation tasks, based on public codes available from https://github.com/HiLab-git/SSL4MIS (assessed on 12 September 2024), and trained it three times with a fixed random seed, without any pretrained weights. During training, the network parameters were updated by the Stochastic Gradient Descent (SGD) optimizer with an initial learning rate of 0.01 and a maximum iteration number of 6000. The learning rate was decayed by a factor of 0.1 every 2500 iterations. The batch sizes were set to 4 and 24 for the LASC and ACDC datasets, respectively, where the number of labeled and unlabeled images was equal. The parameters β and υ were set to 0.001 and 2, respectively. Other parameters were set as follows: $\alpha =\min\left(1-1/t,\;0.99\right)$, K=8, and C=2, following previous configurations for the UA-MT. During training, we randomly cropped the LA region from the LASC dataset with dimensions of 112 × 112 × 80 voxels, resized ACDC slices to 256 × 256 pixels, and augmented these images randomly (e.g., rotation and flip) to avoid over-fitting. In addition, we compared the developed method with four semi-supervised segmentation methods (i.e., MT [14], UA-MT [18], SASSNet [20], and DTC [21]) to demonstrate its effectiveness and accuracy. For a fair comparison, the involved methods were based on the same backbones (i.e., V-Net and U-Net) and trained on two different proportions of labeled and unlabeled images from the training sets (n = 80 and 70) in the LASC and ACDC datasets to demonstrate their segmentation performance and reliance on labeled data. Specifically, 5% (10%) of the images in the training sets were used as labeled data and 95% (90%) of the images were used as unlabeled data for network training. After training, their performances were independently assessed based on the validation sets of the LASC and ACDC datasets, following previous studies [14,17,18].

### 3.3. Segmentation of the LASC Dataset

**Figure 4:**
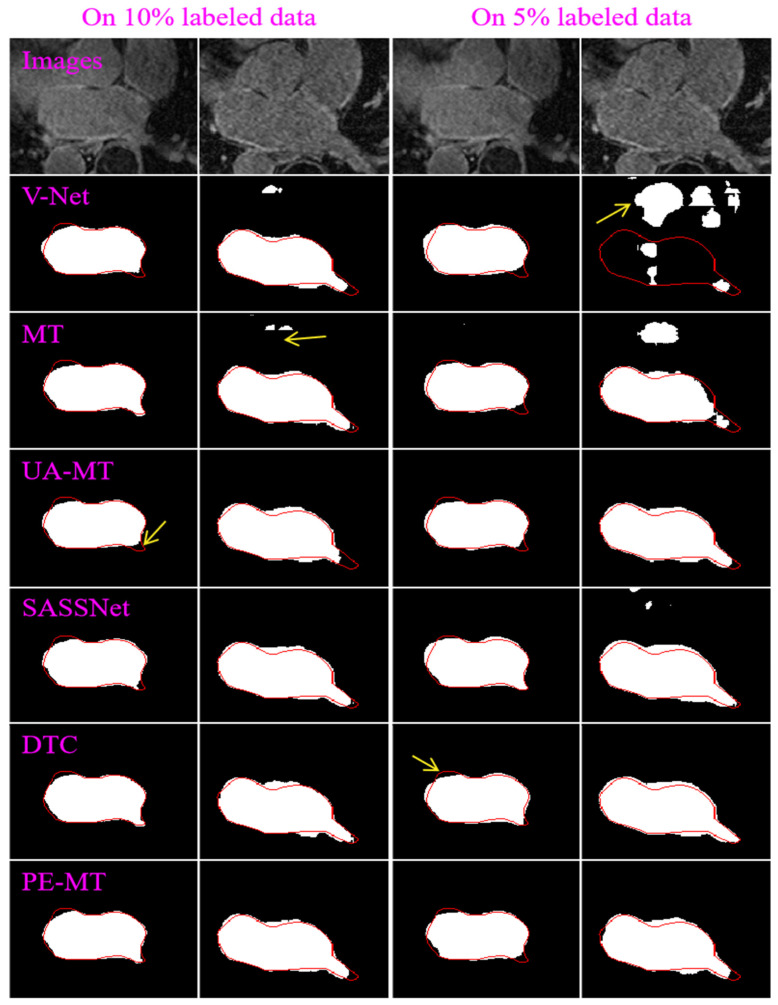
Segmentation results of four given images obtained by the V-Net, MT, UA-MT, SASSNet, DTC, and PE-MT, respectively, which were trained on 10% (in the first two columns) and 5% (in the last two columns) of labeled images from the LASC dataset. The red lines represent the object boundaries of the LA labels, and the yellow arrows indicate the poor segmentation.

Table 1 demonstrates the results of the involved semi-supervised methods based on the V-Net backbone and the validation set of the LASC dataset for the LA segmentation. It can be seen from the results that (1) our developed method obtained an average DSC of 0.8341 and 0.8729 when trained on 5% and 10% labeled data, respectively. It outperformed the MT (0.7916 and 0.8631), UA-MT (0.8080 and 0.8648), SASSNet (0.8137 and 0.8623), and DTC (0.8067 and 0.8679) based on the same backbone and experimental dataset. This showed the advantages of the developed method in image segmentation over the other four semi-supervised methods. (2) All the involved methods had better segmentation performance than V-Net (0.5043 and 0.7610), which was solely trained on the involved labeled images in a fully supervised manner, suggesting the importance of unlabeled images in the semi-supervised learning framework. (3) These semi-supervised methods had an increased segmentation performance when trained on more labeled images and gradually approached the performance of V-Net trained on all the labeled images in a fully supervised manner. Figure 4 illustrates the segmentation results of the involved methods for four different images from the LASC dataset.

### 3.4. Segmentation of the ACDC Dataset

Table 2 shows the results of our developed method based on the U-Net backbone and the validation set for segmenting the RV, Myo, and LV regions from the ACDC dataset in the first experiment. As demonstrated by the results, our developed method had an increased performance in the semi-supervised segmentation framework when trained on more labeled images and could compete with the U-Net in the fully supervised framework. Specifically, our developed method had a average DSC of 0.4166, 0.5635, and 0.6864 for the RV, Myo, and LV, respectively, when trained on 5% labeled data, and 0.6199, 0.7932, and 0.8482 for the three regions when trained on 10% labeled data. It was superior to U-Net for three different objects on average when merely using 5% and 10% labeled data for network training, as shown in Table 2.

**Figure 5:**
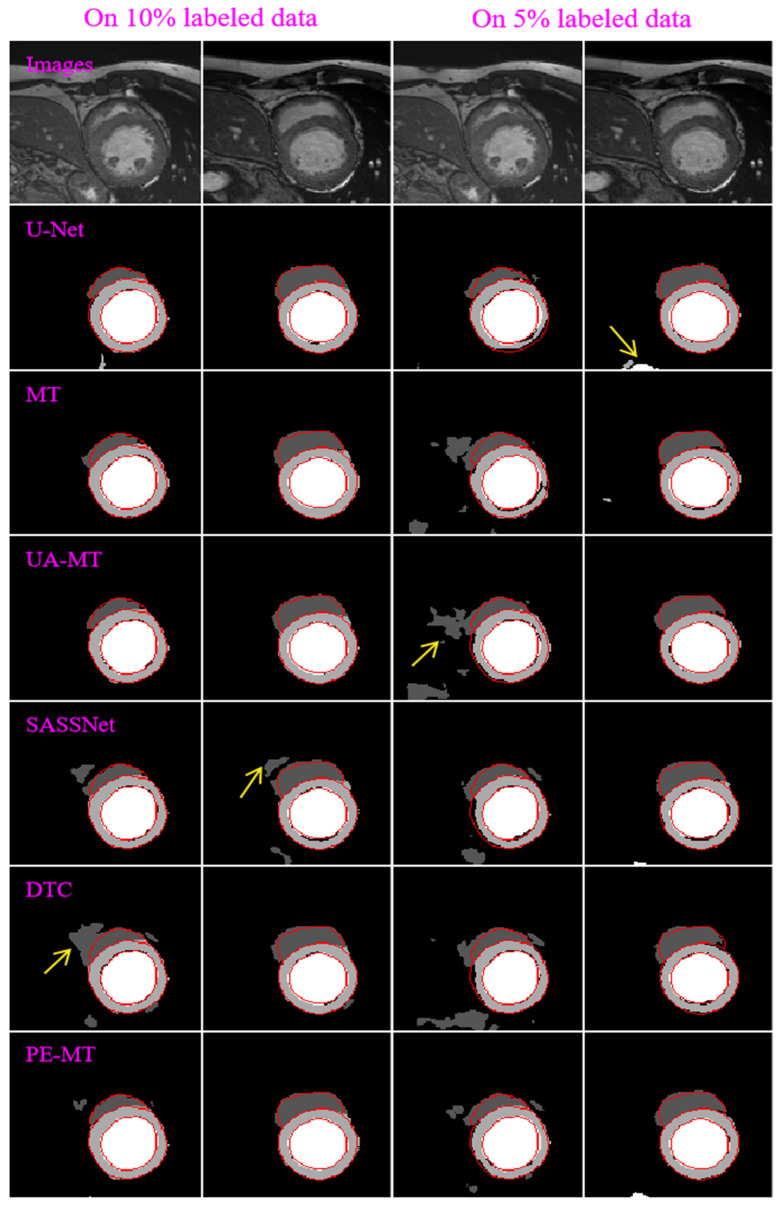
Segmentation results of four different images obtained by the U-Net, MT, UA-MT, SASSNet, DTC, and PE-MT, respectively, using 10% (in the first two columns) and 5% (in the last two columns) labeled images from the ACDC dataset. The red lines represent the ground-truth boundaries, and the yellow arrows indicate the poor segmentation.

Table 3 summarizes the average segmentation results of the involved semi-supervised methods for three different experiments based on the U-Net backbone and ACDC dataset. As shown by the results, these semi-supervised methods had an improved segmentation performance on the validation set of the ACDC dataset when using more labeled images for network training, and they gradually approached the fully supervised results of U-Net trained on all the labeled images. However, they had very different capabilities in extracting three object regions from the ACDC dataset. Specifically, our developed method had an average DSC of 0.5555 and 0.7538 for three different regions (i.e., the LV, Myo, and RV) when trained on 5% and 10% labeled data, respectively. It was superior to the MT (0.5457 and 0.7483) and UA-MT (0.5383 and 0.7385) but inferior to the DTC (0.5601 and 0.7842) and SASSNet (0.5897 and 0.8108) under the same experiment conditions. Figure 5 illustrates the segmentation results of the involved methods for four different images from the ACDC dataset.

### 3.5. Ablation Study

#### 3.5.1. Effect of the pEMA and RUM

**Figure 6:**
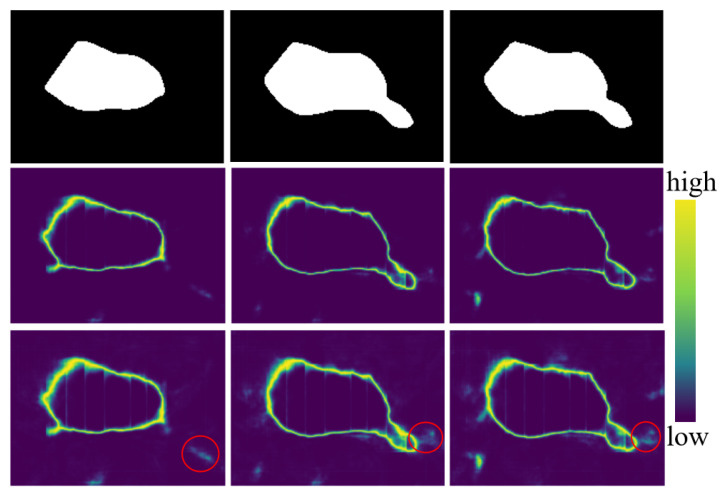
From top to bottom, the labels of three given images and their corresponding uncertainty maps obtained by the RUM and EUM in each row are shown, respectively, where red circle highlighted irrelevant background regions.

Table 4 summarizes the impact of the pEMA and RUM on the performance of the UA-MT for two different segmentation tasks by using the two components to replace the EMA and EUM (note that UA-MT can be viewed as a combination of the EMA, EUM, student, and teacher models, while PE-MT was a variant of UA-MT created by introducing the pEMA and RUM). It can be seen that the UA-MT had a consistently increased average performance for different object regions depicted on the LASC and ACDC datasets when introducing the pEMA and RUM to replace their corresponding original versions (i.e., EMA and EUM). This suggested the effectiveness of our introduced pEMA and RUM, as compared to the EMA and EUM. Figure 6 shows the difference between the RUM and EUM in semi-supervised image segmentation. It can be seen that our introduced RUM can effectively identify and highlight unreliable prediction regions and suppress the adverse impact of background information far away from desirable objects, while the EUM detected lots of background regions, especially those close to object boundary regions.

#### 3.5.2. The Parameters υ and β

Table 5 and Table 6 separately show the impact of the parameters υ and β in RUM and pEMA on the performance of the developed method in two specific segmentation experiments based on the LASC and ACDC datasets. As demonstrated by these results, our developed method achieved better overall performance when setting the parameter υ to 2 for both the LASC and ACDC datasets. Fixing this parameter value of 2, our developed method obtained higher accuracy when setting the parameter β to 0.001 for the same segmentation tasks, as shown in Table 6.

## 4. Discussion

In this paper, we proposed a novel semi-supervised learning method (PE-MT) based on the UA-MT and validated it by extracting multiple cardiac regions from the public LASC and ACDC datasets. The experimental results showed that our developed method can effectively extract desirable object regions by leveraging two available network backbones (i.e., V-Net and U-Net), and it obtained promising segmentation accuracy, owing to the introduction of the pEMA and RUM when trained on 5% (10%) labeled images and 95% (90%) unlabeled ones from the training sets. It was superior to the MT and UA-MT and could compete with the SASSNet and DTC when trained on the same number of labeled and unlabeled images from the LASC and ACDC datasets. Moreover, our methods tended to have increased segmentation accuracy when trained on more labeled and unlabeled images and was able to rapidly process an unseen image at the inference stage (around 1 s).

Our developed method was derived from the UA-MT and superior to it under the same experimental conditions. This was mainly attributed to the introduction of the RUM and pEMA. The RUM had a reasonable capability to accurately identify some regions with high uncertainty in the prediction maps of unlabeled images obtained by the teacher model. By eliminating these prediction regions of high uncertainty, the student and teacher models were able to highlight reliable prediction regions in the calculation of the unsupervised loss and thus improve the prediction accuracy and consistency of the student and teacher models for unlabeled images. This largely enhanced the segmentation potential of the two models and excluded the impact of irrelevant information on the final performance. Moreover, the performance can be further enhanced leveraging the introduced pEMA since it was able not only to provide proper network weights for the teacher model but also to increase the learning flexibility of the student model by adding a random weight perturbation to suppress the coupling effect between the two models. The learning flexibility can to some extent facilitate the detection of various object features and increase the use efficiency of label information.

Despite promising performance, our developed method was inferior to the SASSNet and DTC when extracting three different object regions from the ACDC datasets. This may be due to the fact that (1) our developed method merely employed the V-Net and U-Net to segment desirable objects and did not involve additional network branches or auxiliary learning tasks in image segmentation. In contrast, both the SASSNet and DTC used multiple network branches to simultaneously extract desirable objects and their corresponding signed distance maps in a mutually collaborative manner. This can enhance the learning procedure of specific neural networks due to the introduction of extra network parameters and auxiliary processing tasks and hence improve image segmentation accuracy. (2) V-Net and U-Net had limited learning capability and network parameters (see their structures at https://github.com/HiLab-git/SSL4MIS) (assessed on 12 September 2024) and could not capture enough convolutional features for segmentation purposes when trained on a very small number of labeled images (e.g., three and seven patients’ scans). (3) Labeled images were much less than unlabeled ones in segmenting the cardiac regions from the ACDC dataset, which may have lead to very large data distribution differences (or domain shifts) between the two types of images. These differences made our method subject to relatively severe performance degradation, as compared with the SASSNet and DTC.

Finally, there were some limitations to this study. First, our developed method was merely validated based on the plain network backbones (e.g., V-Net and U-Net), which had relatively limited learning capability, as compared with other deep learning architectures such as Transformers and multi-layer perceptrons (MLPs). This can largely suppress its segmentation performance and clinical application potential. Second, only a few data augmentation schemes (e.g., rotation and flip) were used in the segmentation experiments, potentially making our developed method have low accuracy in segmenting different medical images with varying modalities. Third, both the LASC and ACDC datasets contained a very small number of images and were further split into training and testing sets. This may have caused our developed method to be unable to capture various convolutional features associated with target objects, and thus, it underwent a rapid performance degradation when labeled images were reduced in the training set. Last but not least, our developed method was not validated for dynamical image segmentation [29], which aims to process multiple different images at multiple different instances of time or in multiple videos [30,31]. The incomplete performance validation not only limits the potential applications of the developed algorithm but also restricts its popularization. Despite these limitations, our model achieved promising segmentation performance on two public image datasets and surpassed the UA-MT under the same experimental configuration.

## 5. Conclusions

We developed a novel semi-supervised learning method (termed PE-MT) for accurate image segmentation based on a small number of labeled data and a large number of unlabeled data. Its novelty lies in the introduction of the pEMA and RUM and their integration with the available UA-MT. The pEMA extended the original EMA and added an adaptive weight perturbation to the student model in order to enhance its learning flexibility and effectiveness, while the RUM alleviated the drawbacks of the EUM in the UA-MT via a quantitative uncertainty formula and was used to filter out some prediction regions with high uncertainty. Extensive segmentation experiments on the public LASC and ACDC datasets demonstrated that the developed method was able to effectively extract desirable objects when trained on a small number of labeled images and a large number of unlabeled images and outperformed the MT and UA-MT under the same experimental configuration.

## Figures and Tables

**Table 1:** The LA segmentation results on the validation set in terms of the average DSC, JAC, HD and ASD, leveraging the involved methods, which were trained on different proportions of labeled data and unlabeled images from the training set of the LASC dataset.

Method	Number of Images		Metrics			
	Labeled	Unlabeled	DSC	JAC	HD	ASD
V-Net	80	0	0.9178	0.8485	4.7179	1.5867
V-Net	4	0	0.5043	0.3972	36.3690	11.0264
MT	4	76	0.7916	0.6631	24.8149	7.0991
UA-MT	4	76	0.8080	0.6868	21.7672	6.5760
SASSNet	4	76	0.8137	0.6924	27.8814	8.0149
DTC	4	76	0.8067	0.6856	26.6678	7.5836
PE-MT	4	76	0.8341	0.7225	18.9836	5.0198
V-Net	8	0	0.7610	0.6527	26.9073	4.8357
MT	8	72	0.8631	0.7612	17.9738	4.5731
UA-MT	8	72	0.8648	0.7638	16.7100	4.3400
SASSNet	8	72	0.8623	0.7612	13.1187	3.7583
DTC	8	72	0.8679	0.7692	11.6410	3.3986
PE-MT	8	72	0.8729	0.7758	13.1082	3.8202

**Table 2:** The cardiac segmentation results on the validation set in terms of the average DSC, JAC, HD, and ASD, leveraging the developed method and U-Net in the first experiment, which were trained on different proportions (i.e., 5% and 10%) of labeled data and unlabeled images from the training set of the ACDC dataset.

Dataset	Method	Number of Images		Metrics			
		Labeled	Unlabeled	DSC	JAC	HD	ASD
RV	U-Net	3	0	0.3930	0.2836	63.1196	30.3970
	PE-MT	3	67	0.4166	0.2998	62.2174	26.3911
	U-Net	7	0	0.6323	0.5096	24.0267	8.4186
	PE-MT	7	67	0.6199	0.4994	18.4767	6.1613
Myo	U-Net	3	0	0.5145	0.3983	20.1485	6.9656
	PE-MT	3	67	0.5635	0.4432	18.5294	7.0502
	U-Net	7	0	0.7943	0.6704	8.6746	2.2788
	PE-MT	7	63	0.7932	0.6675	9.7917	2.9752
LV	U-Net	3	0	0.5607	0.4430	56.9506	21.5382
	PE-MT	3	67	0.6864	0.5819	38.3050	13.7716
	U-Net	7	0	0.8403	0.7427	29.9437	8.5729
	PE-MT	7	63	0.8482	0.7511	34.2763	9.3469

**Table 3:** The cardiac segmentation results on the validation set in terms of the average DSC, JAC, HD, and ASD, leveraging the involved semi-supervised methods and U-Net, which were trained on different proportions of labeled data and unlabeled images from the training set of the ACDC dataset for three experiments.

Method	Number of Images		Metrics			
	Labeled	Unlabeled	DSC	JAC	HD	ASD
U-Net	70	0	0.8807	0.7936	6.4722	1.8963
U-Net	3	0	0.4894	0.3750	46.7396	19.6336
MT	3	67	0.5457	0.4333	43.9185	17.3452
UA-MT	3	67	0.5383	0.4272	41.3736	16.0410
SASSNet	3	67	0.5897	0.4752	23.3788	8.5670
DTC	3	67	0.5601	0.4511	26.4061	11.1162
PE-MT	3	67	0.5555	0.4416	39.6839	15.7376
U-Net	7	0	0.7556	0.6409	20.8817	6.4234
MT	7	63	0.7483	0.6340	20.2368	5.6540
UA-MT	7	63	0.7385	0.6199	21.0633	5.9992
SASSNet	7	63	0.8108	0.7074	12.3803	3.6314
DTC	7	63	0.7842	0.6842	10.1061	3.0190
PE-MT	7	63	0.7538	0.6393	20.8482	6.1611

**Table 4:** Performance of the UA-MT trained on 10% labeled data and 90% unlabeled data from the training set in the LASC and ACDC datasets by using the pEMA and RUM to replace the EMA and EUM, respectively.

Dataset	Method	Number of Images		Metrics			
		Labeled	Unlabeled	DSC	JAC	HD	ASD
LASC	UA-MT	8	72	0.8648	0.7638	16.7100	4.3400
	UA-MT + RUM	8	72	0.8724	0.7753	14.4020	3.7612
	UA-MT + RUM + pEMA	8	72	0.8729	0.7758	13.1082	3.8202
ACDC	UA-MT	7	63	0.7385	0.6199	21.0633	5.9992
	UA-MT + RUM	7	63	0.7429	0.6237	25.2195	7.3287
	UA-MT + RUM + pEMA	7	63	0.7538	0.6393	20.8482	6.1611

**Table 5:** Performance of the PE-MT when setting different values for the parameter υ in the RUM for two different segmentation tasks.

Dataset	υ	Number of Images		Metrics			
		Labeled	Unlabeled	DSC	JAC	HD	ASD
LASC	1	8	72	0.8615	0.7586	16.4457	3.9698
	2	8	72	0.8724	0.7753	14.4020	3.7612
	3	8	72	0.8631	0.7623	14.7983	3.7027
ACDC	1	7	63	0.7229	0.6109	21.0683	6.5155
	2	7	63	0.7429	0.6237	25.2195	7.3287
	3	7	63	0.7297	0.6142	25.6428	7.4772

**Table 6:** Performance of the PE-MT when setting different values for the parameter β in pEMA for two different segmentation tasks.

Dataset	β	Number of Images		Metrics			
		Labeled	Unlabeled	DSC	JAC	HD	ASD
LASC	0.005	8	72	0.7440	0.6084	21.5900	5.4993
	0.001	8	72	0.8729	0.7758	13.1082	3.8202
	0.0005	8	72	0.8590	0.7550	17.7567	4.6438
	0.0001	8	72	0.8630	0.7616	18.6198	4.5289
ACDC	0.005	7	63	0.7026	0.5746	31.8241	11.4408
	0.001	7	63	0.7538	0.6393	20.8482	6.1611
	0.0005	7	63	0.7248	0.6077	22.9209	6.6283
	0.0001	7	63	0.7449	0.6246	25.7077	7.4602

## Data Availability

The data presented in this study are available upon request from the corresponding authors.
